# Risk Stratification for Autoimmune Hemolytic Anemia in Children With Pediatric Transfusion-Dependent Thalassemia

**DOI:** 10.7759/cureus.71901

**Published:** 2024-10-20

**Authors:** Arooj Khan, Faizan Sadiq, Shaista Azeem Khan, S Mohsin Ali Shah, Misbah Ullah Khan, Muhammad Khalid Khan, Anam Zaman, Abdur Rehman, Faran Sadiq, Muhammad Rahat Jan

**Affiliations:** 1 Department of Paediatrics, Khyber Medical College and Teaching Hospital, Peshawar, PAK; 2 Department of Accident and Emergency, Lady Reading Hospital, Peshawar, PAK

**Keywords:** autoimmune hemolytic anemia (aiha), coombs test, pediatric hematology, risk factors, transfusion-dependent thalassemia (tdt)

## Abstract

Objective

This study aimed to identify the risk factors for autoimmune hemolytic anemia (AIHA) in children with transfusion-dependent thalassemia (TDT).

Methodology

This cross-sectional study was conducted at the Department of Child Health, Khyber Teaching Hospital, Peshawar, Pakistan, from March 25, 2024, to September 25, 2024. A total of 300 patients under 18 years of age, diagnosed with TDT, were included in the study. Relevant demographic, clinical, and laboratory investigations were conducted for each patient, with AIHA diagnosed based on the Coombs test. Risk factors, including demographic data, disease characteristics, transfusion history, clinical factors, and laboratory parameters, were recorded and analyzed. The significance of these risk factors was assessed by using the chi-square test for categorical variables and the independent t-test for continuous variables, comparing Coombs test-compatible and incompatible patients. A p-value of less than 0.05 was considered statistically significant.

Results

The mean±SD age of participants in this study was 6.82±4.2 years with an age range of one to 16 years. The male gender was 56.33%. The results of the Coombs test showed that 48.66% of these children had developed AIHA.

The comparison between incompatible and compatible patients at Coombs test showed that age of patients (5.87±3.93 vs. 7.68±4.28 years, p = 0.00), age at the time of diagnosis (2.21±0.99 vs. 4.64±3.03 years, p = 0.00), age at the time of first infusion (3.05±1.28 vs. 5.49±3.2 years, p = 0.00), transfusion interval < 3 weeks (48.6% vs. 37%, p = 0.04) and duration of transfusion (2.87±3.2 vs. 2.21±1.93 years, p = 0.03) were the significant risk factors in the development of AIHA.

Conclusion

The patient’s age, age at diagnosis, age at the time of first transfusion, the transfusion interval, and the duration of blood transfusion are the significant risk factors influencing the development of AIHA.

## Introduction

Thalassemia refers to a diverse set of inherited blood disorders that impact the genes responsible for hemoglobin production, leading to impaired red blood cell formation [1]. Beta (β)-thalassemia is a blood disorder characterized by reduced or absent production of beta-globin chains, a crucial component of hemoglobin. This genetic condition leads to decreased red blood cell production and anemia, ranging from mild to severe. Reduced hemoglobin production causes anemia at a young age and often necessitates regular blood transfusions to maintain adequate hemoglobin levels [1,2]. This transfusion-dependent thalassemia (TDT) encompasses a range of thalassemia severities in which patients require regular blood transfusions at intervals of two to six weeks to maintain their hemoglobin levels between 9 g/dL and 11.5 g/dL [3].

Historically, β-thalassemia has been more prevalent in specific areas, including the Mediterranean, the Middle East, and Southeast Asian countries. However, due to large population movements, this condition is becoming increasingly common in other parts of the world, such as Northern Europe and North America [4]. The WHO's 2008 report indicated an annual global incidence of over 40,000 β-thalassemia births, with 25,500 requiring transfusions. These outcomes are also observed to vary significantly between high- and low-income countries. From 1990 to 2021, the global thalassemia burden has decreased overall but increased among the elderly. Changing patterns in the geographical and population-based distribution of thalassemia cases demonstrate the effects of healthcare strategies and community health initiatives on the management and outcomes of the disease [5]. In Pakistan, the prevalence of the β-thalassemia trait is estimated at 5%-7% of the population, translating to over 10 million carriers nationwide. Annually, approximately 5,000 new cases of β-thalassemia major are diagnosed among Pakistani children [6].

Patients with TDT receiving multiple blood transfusions are at risk of developing both autoantibodies (autoimmunization when chronic immune activation and inflammation trigger the production of autoantibodies against the patient’s own red blood cells) and alloantibodies (alloimmunization, when the immune system becomes sensitized to foreign antigens on transfused red blood cells, leading to the production of alloantibodies) against donor erythrocytes. Furthermore, immunological dysregulation may result from prolonged transfusion-related iron overload. The incidence of alloimmunization in thalassemia patients varies globally, ranging from 4% to 50% of cases [7].

Transfusion reactions increase with high hemolysis rates due to the shortened lifespan of erythrocytes, escalating the need for more frequent transfusions. This strains the blood supply at blood banks and transfusion centers. Consequently, treating AIHA becomes necessary, leading to higher costs and longer hospital stays. Alloimmunization and the formation of alloantibodies against erythrocyte antigens complicate cross-matching and reduce the in vivo survival of transfused erythrocytes. This causes transfusion reactions and difficulties in providing safe transfusions, thereby increasing the risk of iron overload [8].

There is no consensus on the risk factors for AIHA in pediatric TDT patients. Studies have mentioned a list of risk factors associated with AIHA in TDT patients, which include gender, age at diagnosis, age at the first transfusion, transfusion interval, blood type, serum ferritin levels, pre-transfusion hemoglobin level, history of splenectomy, and transfusion volume [9-11].

However, some other studies have found no link between the development of alloantibodies and various patient factors, like age, sex, hemoglobin concentrations, quantity of blood units transfused, or whether the patient had undergone splenectomy [12].

Early identification of high-risk patients is essential for implementing preventive strategies and enhancing outcomes. Thus, the purpose of this study was to identify the risk factors for autoimmune hemolytic anemia (AIHA) in children with TDT.

## Materials and methods

Study design, period, and participants

This cross-sectional study was conducted at the Department of Child Health, Khyber Teaching Hospital, Peshawar, Pakistan, from March 25, 2024, to September 25, 2024, over a period of six months. The sample size was calculated using the following assumptions: prevalence of AIHA when the first transfusion was performed at <3 years = 74.10%, precision = 5.00%, with a 95% confidence interval and an infinite population size, resulting in an estimated sample size of 295 patients [13]. A total of 300 patients under 18 years of age, reporting to the department, diagnosed with TDT, and having received at least 10 transfusions, were included in this study through consecutive sampling. Patients with comorbid diseases affecting the immune system (such as systemic lupus erythematosus or cancers) and those currently taking or with a history of corticosteroid or immunosuppressant drug use were excluded from the study.

Study procedure and data collection

All relevant demographic, clinical, and laboratory investigations were conducted for each patient and recorded using a standardized format. The diagnosis of AIHA was confirmed based on compatibility with the Coombs test. The study recorded risk factors such as demographic data (age, gender), disease characteristics (age at TDT diagnosis, duration of transfusion dependency), transfusion history (age at first transfusion, transfusion interval), clinical factors (splenectomy status, nutritional status), and laboratory parameters (blood type, average pre-transfusion hemoglobin levels [g/dL], serum ferritin levels (μg/L)).

Statistical analysis

Data entry and analysis were performed using the IBM SPSS Statistics software, version 25 (IBM Corp., Armonk, NY). Quantitative parameters were presented as mean ± standard deviation, while qualitative parameters were expressed as frequencies and percentages. The significance of these risk factors was assessed by using the chi-square test for categorical variables and the independent t-test for continuous variables, comparing Coombs test-compatible and incompatible patients. A p-value of less than 0.05 was considered statistically significant.

## Results

The mean±SD of age in this study was 6.82±4.2 years with an age range of one to 16 years. The male gender was 56.33% of the total population. The details of the demographics and medical history of the study group are shown in Table 1.

**Table 1:** Demographics and clinical history of the study group

Demographics		
Age (Mean±SD) (years)		6.82±4.2
Gender	Male n (%)	169 (56.33)
	Female n (%)	131 (43.66)
Clinical history		
Age at diagnosis (Mean±SD) years		3.46±2.58
Age at first transfusion (Mean±SD) (years)		4.3±2.74
Duration of transfusion (Mean±SD) (years)		2.51±2.66
Transfusion interval (week)	< 3	138 (46)
	≥ 3	162 (54)
Splenectomy n (%)		46 (15.33)
Blood group	A n (%)	62 (20.66)
	B n (%)	108 (36)
	O n (%)	75 (25)
	AB n (%)	55 (18.33)
Malnutrition n (%)		161 (53.66)
Hemoglobin/transfusion (g/dL)	< 9 n (%)	298 (99.33)
	≥ 9 n (%)	2 (0.66)
Ferritin levels (ug/L)	≥2500 n (%)	183 (61)
	< 2500 n (%)	117 (39)
Length of transfusion period	< 10 n (%)	297 (99)
	≥10 n (%)	3 (1)

The results of the Coombs test showed that 48.66% of these children had developed AIHA (Table 2).

**Table 2:** Results of the Coombs test

Coomb’s test	
Compatible n (%)	154 (51.33)
Incompatible n (%)	146 (48.66)

The comparison of risk factors between patients who were compatible or incompatible with the Coombs test showed that the age of patients, age at the time of diagnosis, age at the time of first transfusion, transfusion interval, and duration of infusion were the significant risk factors for the development of AIHA in pediatric patients suffering from TDT (Table 3).

**Table 3:** Comparison of the risk factors between patients who were compatible and incompatible with the Coombs test

Risk factors		Compatible (n=154)	Incompatible (n=146)	p-value
Gender	Male n (%)	87 (56.5)	82 (56.2)	0.95
	Female n (%)	67 (43.5)	64 (43.8)	
Age (Mean±SD) (years)		7.68±4.28	5.87±3.93	<0.05
Age at diagnosis (Mean±SD) (years)		4.64±3.03	2.21±0.99	<0.05
Age at 1st transfusion (Mean±SD) (years)		5.49±3.2	3.05±1.28	<0.05
Transfusion interval	< 3 week n (%)	67 (37)	71 (48.6)	<0.05
	≥3 week n (%)	97 (63)	75 (51.4)	
Duration of infusion (Mean±SD) (years)		2.21±1.93	2.87±3.2	<0.05
Splenectomy n (%)		22 (14.3)	24 (16.44)	0.9
Malnutrition n (%)		89 (57.8)	72 (49.32)	0.14
Blood group	A n (%)	29 (18.8)	33 (22.6)	0.65
	B n (%)	60 (39)	48 (33)	
	O n (%)	38 (24.7)	37 (25.34)	
	AB n (%)	26 (16.9)	29 (20)	
Hemoglobin/transfusion (g/dL)	< 9	153 (99.35)	145 (99.32)	0.97
	≥ 9	1 (0.65)	1 (0.68)	
Ferritin levels (ug/L)	< 2500	56 (36.36)	61 (41.8)	0.92
	≥2500	98 (63.64)	85 (58.2)	

## Discussion

Due to the importance of AIHA in pediatric patients, the subject of risk factor identification has been discussed by studies in various study designs and various groups of patients. Khaled et al. mentioned that the development of AIHA is associated with several risk factors, including previous alloimmunization, diagnosis of β-thalassemia intermedia, splenectomy history, early stages of transfusion therapy, genetic predisposition, detection of polyspecific autoantibodies, and AB blood type [14].

A study conducted in 2023 working on the risk factors for alloimmunization found no significant link between the risk of alloimmunization and factors such as gender, age, genotype variant, total number of transfusions, or the duration of transfusion [15]. Dhawan et al. reported the incidence of autoantibodies development as high as 28.2%. The study indicated that patients who received more transfusions were more prone to develop autoantibodies compared to those who received fewer transfusions (p = 0.001) [7].

A study conducted in Thailand reported the incidence of autoantibody development as 15.6%. Significant factors associated with alloimmunization were transfusion interval ≤ 4 weeks (p = 0.03) and initiating blood transfusions at age ≥ 3 years. The presence of splenomegaly and receiving more frequent and larger volume transfusions were other significant risk factors [16].

A Moroccan research investigation worked on the frequency and risk factors of red blood cell alloimmunization in individuals with beta-thalassemia. The research examined potential risk factors for alloimmunization, including patient demographics, transfusion history and frequency, splenectomy status, sequestration events, and the presence of autoantibodies. The findings revealed that the development of autoantibodies (42.85% vs. 8.21%, with P < 0.001), blood transfusions occurring < 3 weeks (71.43% vs. 35.61%, with p = 0.01), and gender (64.29% females vs. 35.71% males, p = 0.01) were correlated with an increased likelihood of AIHA [17].

A study on the risk factors for AIHA in pediatric patients with TDT conducted in Indonesia worked on the factors including age, age at diagnosis, age at first transfusion, transfusion interval, duration of transfusion, history of splenectomy, blood type, nutritional status, average hemoglobin level per transfusion, ferritin level, and the annual volume of blood transfusions. The study shared that age at the first transfusion (p = 0.004) and interval < 3 weeks (0.021) were the most significant risk factors for AIHA reported in these study patients [13].

The results of our study are in line with the studies discussed above and provide a good insight into the risk factors for AIHA in pediatric patients [7,13,16,17]. Current age, age at diagnosis, and age at first infusion reflect disease duration, cumulative transfusion exposure, and onset of regular transfusions, and all these potentially affect immune system development. Transfusion intervals <3 weeks indicate frequent antigen exposure, possibly elevating immune response risk. Infusion duration may impact blood processing and immune reactivity. Collectively, these factors characterize the patient's transfusion burden, significantly affecting AIHA risk in children with TDT. By taking these risk factors into account, clinicians can take extra measures for at-risk patients, thereby minimizing the incidence.

Limitations

The limitations of this study are that it was conducted at a single center and had a cross-sectional design, which restricts the ability to determine causal relationships between the identified risk factors and the development of AIHA in TDT patients. Future research employing longitudinal or interventional designs would provide a more comprehensive understanding of causative factors and clinical outcomes in this patient population.

## Conclusions

The results of this study show that the patient’s age, age at diagnosis, age at the first transfusion, the transfusion interval, and the duration of blood infusion were the significant risk factors influencing the development of AIHA in pediatric patients suffering from TDT. This study provides data for pediatricians to keep in consideration these risk factors while managing the treatment of children with AHA.
